# Supplementary material for: Differential targeting of human pyroptotic caspase-5 and caspase-4 by Shigella OspC2 and OspC3
Source: mBio. 2026 Feb 18;17(3):e03855-25. doi: 10.1128/mbio.03855-25 (PMC12977621; doi:10.1128/mbio.03855-25)
Supplement: Supplemental Material — Figures S1-S6; Tables S1-S3. [file mbio.03855-25-s0001.pdf]

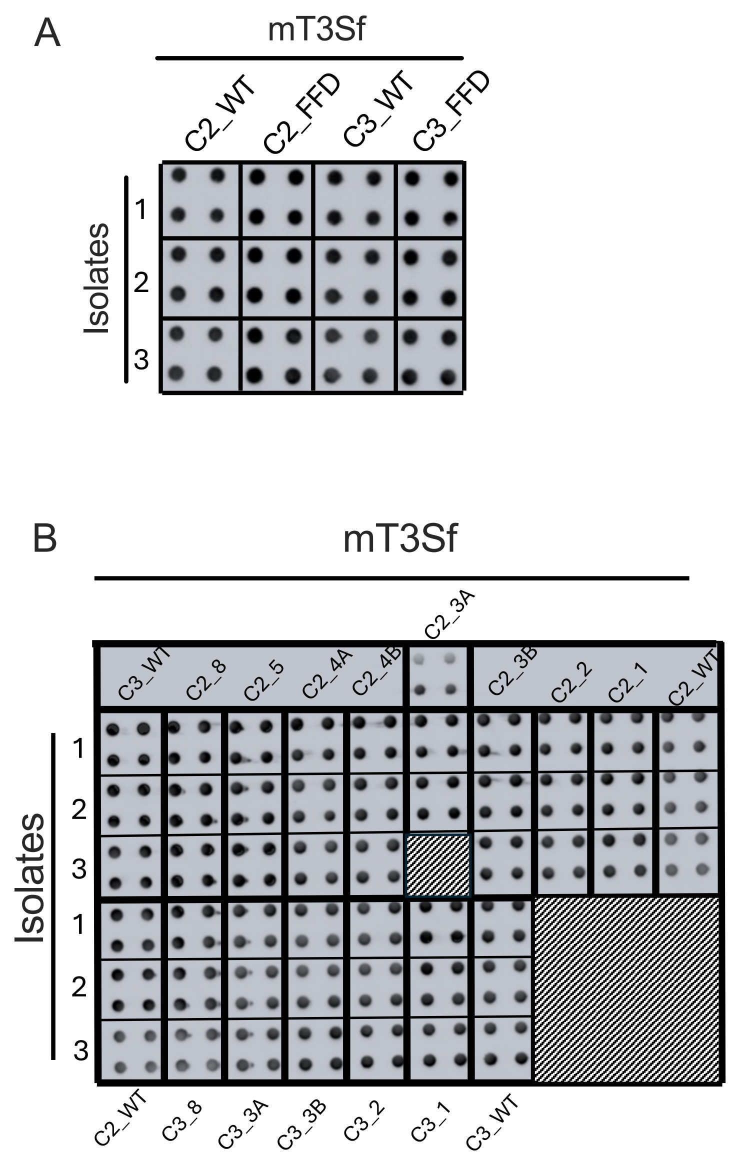

Fig. S1 OspC variants are secreted at levels equivalent to WT effectors. (A-B) Solid plate secretion assay of mT3Sf expressing designated OspC variants. Blots were probed with anti-FLAG antibody. Each set of four spots originated from a single isolate.

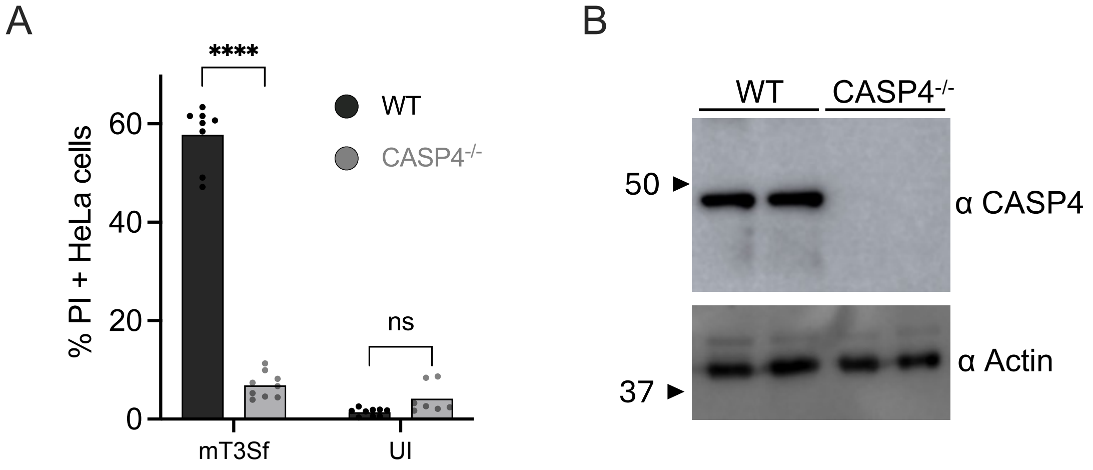

Fig. S2 Infection with mT3Sf triggers CASP4-dependent pyroptosis of epithelial cells. (A) IFNγ-primed WT or CASP4$^{-/-}$ HeLa cells were infected with mT3Sf at an MOI of 5. One-hour later, gentamicin, PI and Hoechst were added to the media. After Two hours, the cells were imaged and the percentage of PI+ cells quantified. Each infection condition included at least three technical repeats and that shown is representative of at least 3 independent assays. (B) Immunoblots of whole cell lysates two independent CASP4$^{-/-}$ HeLa cells probed with designated antibodies.

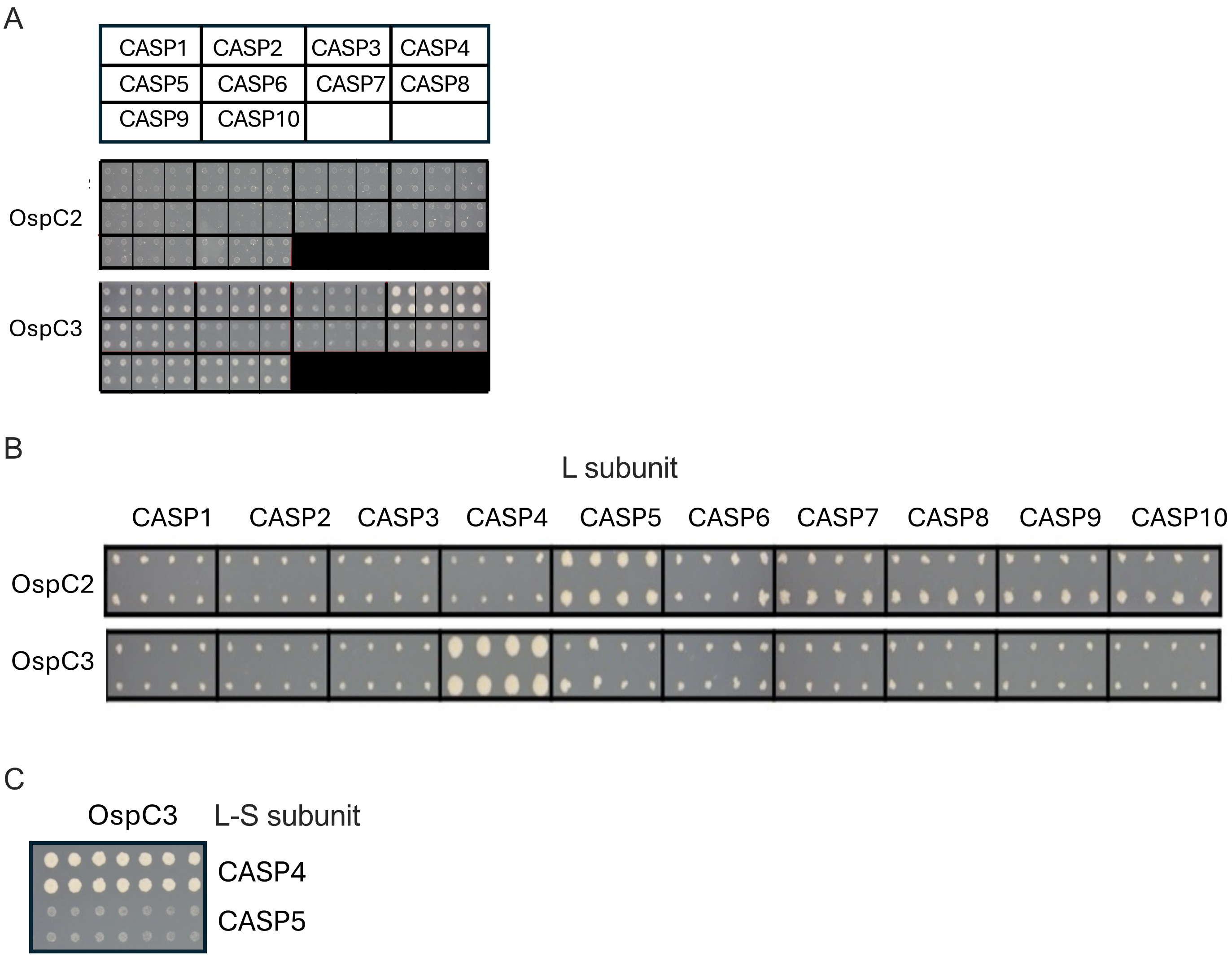

Fig. S3 OspC2 and OspC3 specifically interact with and target CASP5 and CASP4, respectively. (A-C) Y2H assays were performed to assess the interactions of OspC2 and OspC3 with full-length human caspases 1-10 (A), their respective L domains (B), and OspC3 with the L-S domain of CASP5 (C). Images shown were obtained after seven days of incubation on selective media.

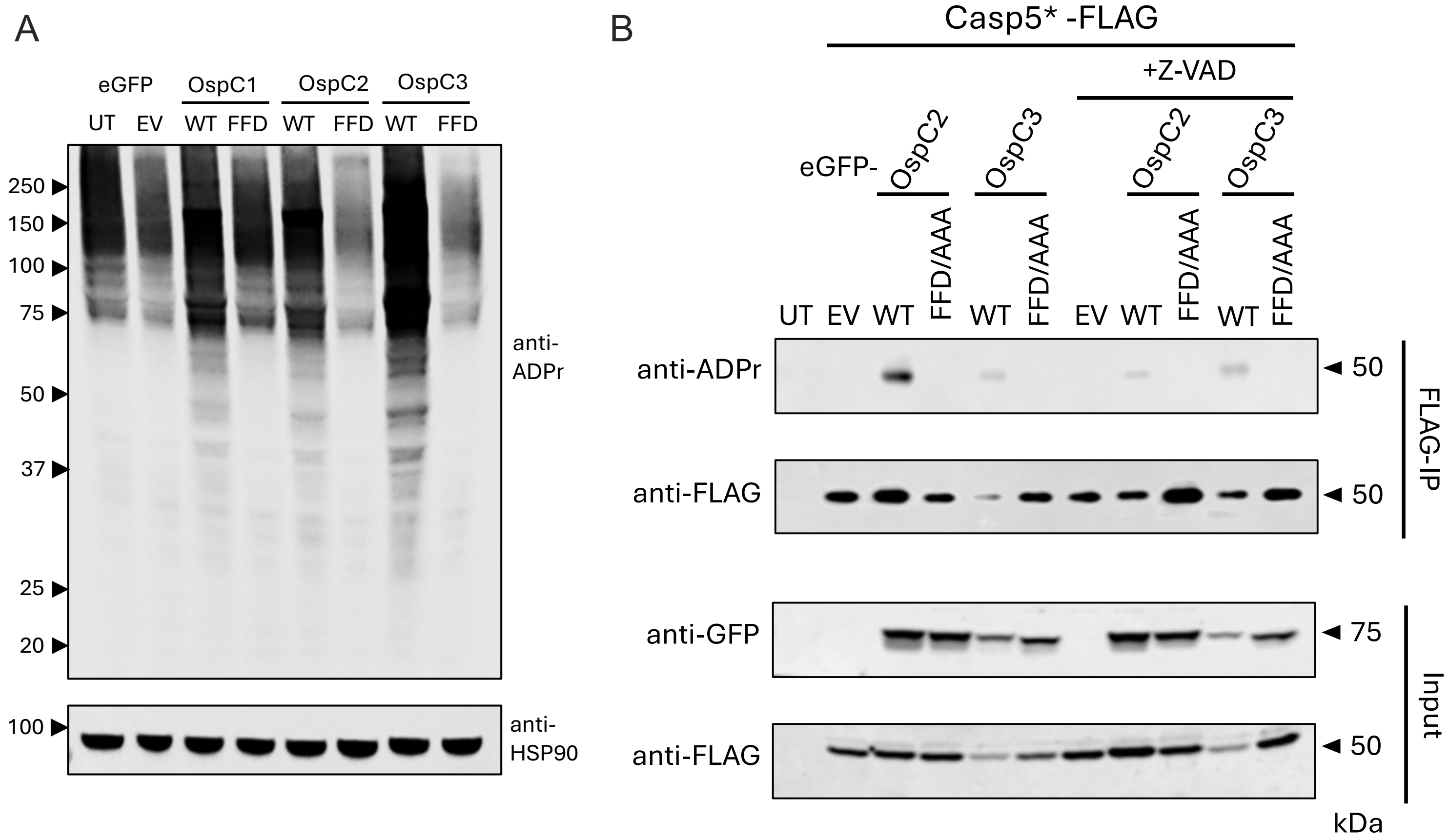

Fig. S4 ADP-riboxanation profiling of OspC variants. (A) HEK293 cells were transfected with a plasmid that expresses a WT or catalytically dead GFP-OspC variant. After 48h, cells were lysed and the whole cell lysates were probed with the designated antibodies. (B) HEK293 cells were co-transfected with plasmids that express the designated GFP-OspC variant and one the expresses 3xFLAG tagged catalytically dead CASP5 (B). After 24h, ZVAD was added to the media. After an additional 48h the cells were lysed, and the FLAG-tagged caspases were immunoprecipitated. Immunoblots of the input and immunoprecipitated fractions were probed with the designated antibodies. Panel (A) shows results from three experiments; panel (B) reports ZVAD effect from one experiment.

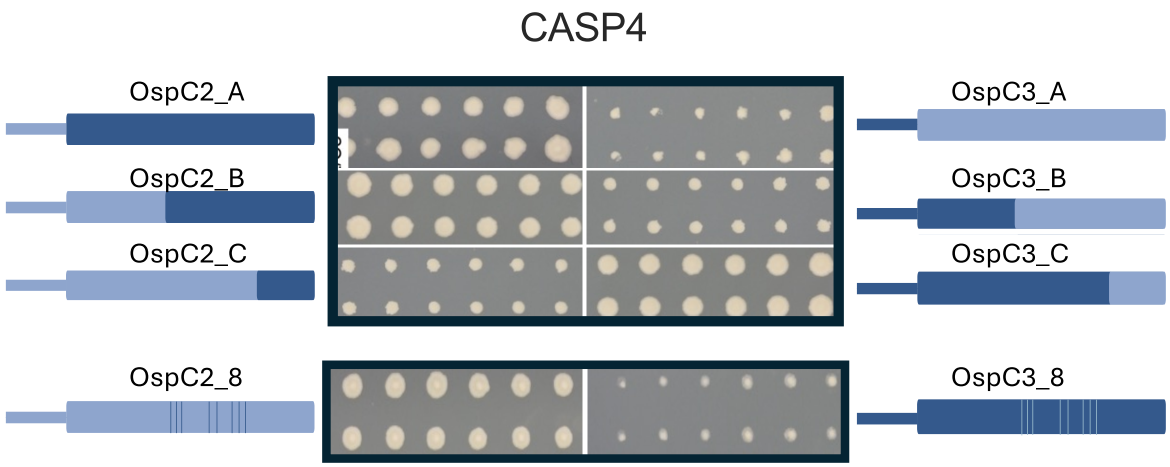

Fig. S5 Identification of OspC chimeras that bind to full-length CASP4. (A) Y2H assays were performed to assess the interactions between OspC chimeras and full-length CASP4. Images obtained after 7 days of growth on selective media.

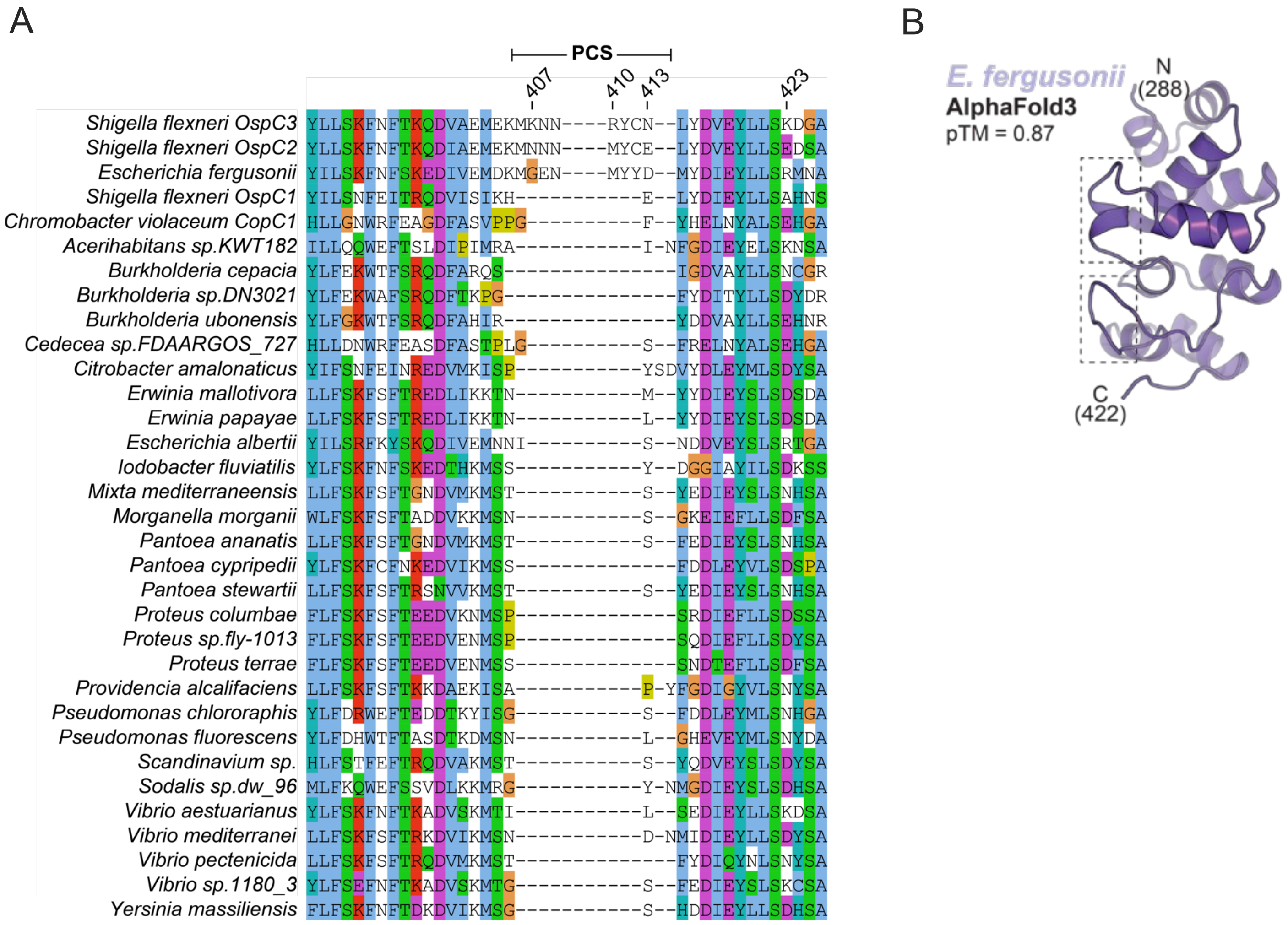

Fig. S6 Alignment of ARDs of OspC homologs. (A) Alignment of upstream region of ARD domains of OspC homologs identified via a BLAST search with full length OspC3. (B) AlphaFold3 prediction of the structure of the ARD of *E. fergusonii.* The color scheme follows standard CLUSTAL coloring, whereby conserved hydrophobic residues are indicated in blue, basic residues in red, acidic residues in magenta, polar residues in green, cysteines in pink, glycines in orange, prolines in yellow, and aromatics in cyan.

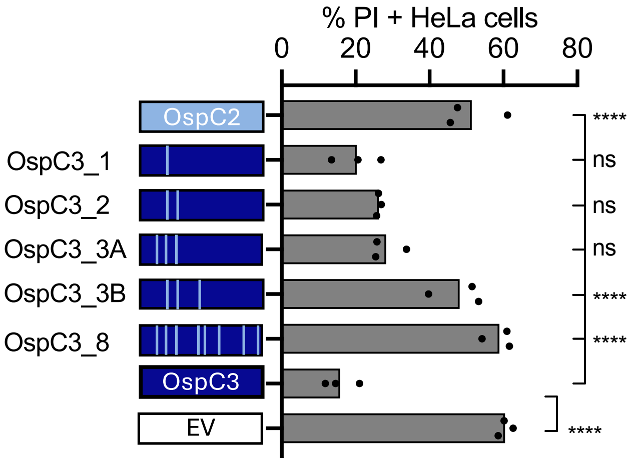

Fig. S7 Characterization of the ability of OspC3 variants to suppress mT3Sf-triggered cell death IFNγ-primed HeLa cells were infected with designated mT3Sf strain at MOI of 5. One-hour post-infection, gentamicin, PI and Hoescht were added to the media. After an additional 2h, the cells were imaged and the percentage of PI+ cells determined. Each infection condition included at least three technical repeats and that shown is representative of at least 3 independent assays.

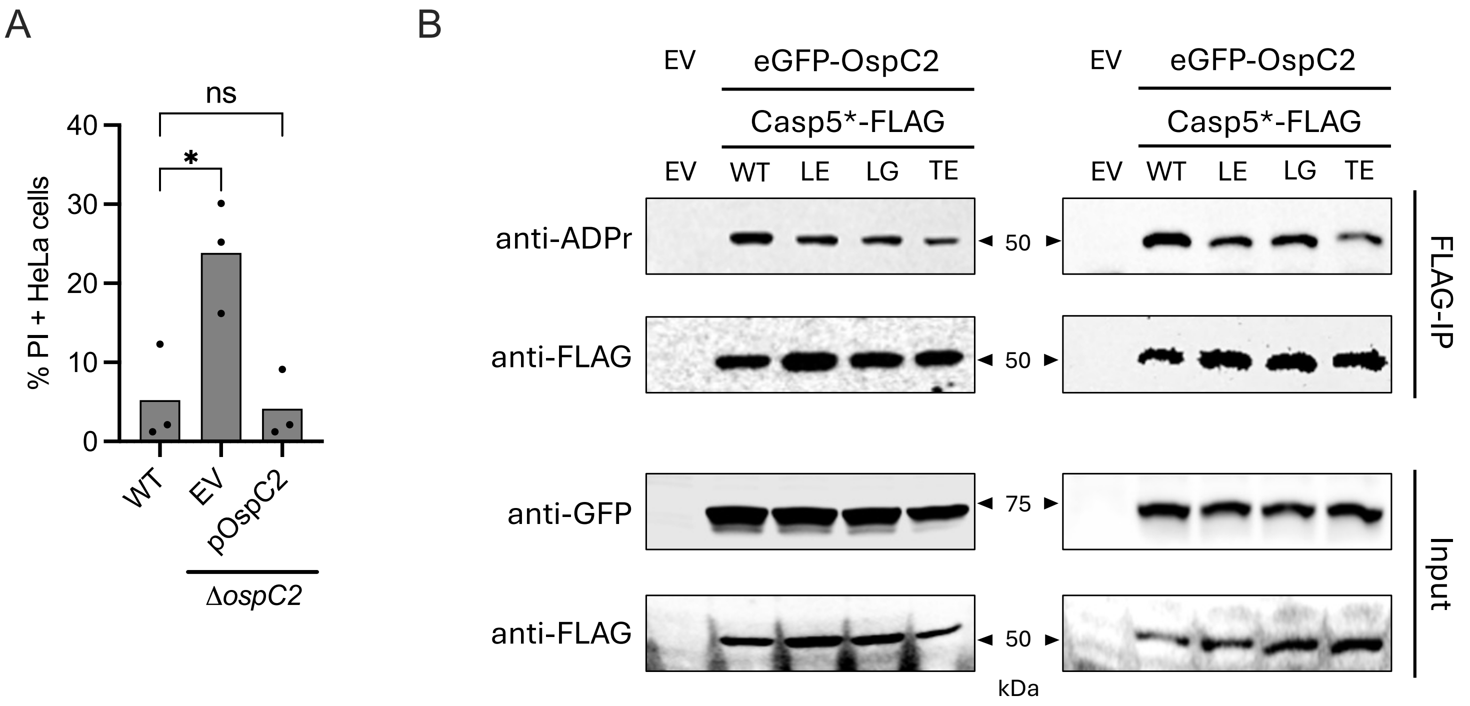

Fig. S8 OspC2 modifies CASP5 through recognition of positively selected residues in an exposed CASP5 α-helix. (A) IFNγ-primed HeLa cells were infected with designated WT, Δ*ospC3* or Δ*ospC3* *Shigella* at an MOI 10. Thirty minutes post-infection gentamicin, PI and Hoescht were added to the media. After an additional 2h, the cells were imaged and the percentage of PI+ cells determined. Statistical significance when indicated was assessed by one-way ANOVA with Tukey's post hoc test. *P < 0.05, **P < 0.01, ****P < 0.0001, ns = nonsignificant. (B) HEK293 cells were co-transfected with plasmids that express GFP-OspC2 and the indicated 3xFLAG tagged catalytically dead full-length variant of CASP5. After 24h, the cells were lysed and the FLAG-tagged CASP5s were immunoprecipitated. Immunoblots of the input and immunoprecipitated fractions were probed with the designated antibodies. The blots shown are representative of two experimental repeats.

TABLE S1 Gateway entry plasmids

	insert origin	source
pDONR221 (KanR)		Invitrogen
pDONR223 (SpecR)		Invitrogen
**Full length caspases**		
pENTR223 CASP1		PlasmID
pENTR223 CASP2		PlasmID
pENTR223 CASP3		PlasmID
pENTR221 CASP4		PlasmID
pENTR221 CASP5		PlasmID
pENTR223 CASP6		PlasmID
pENTR223 CASP7		PlasmID
pENTR221 CASP8		PlasmID
pENTR221 CASP9		PlasmID
pENTR 223 CASP10		PlasmID
**Caspase 4 domains**		
pENTR221 CASP4 CARD	PCR	this study
pENTR221 CASP4 L	PCR	this study
pENTR221 CASP4 S	PCR	this study
pENTR221 CASP4 LS	PCR	this study
**Caspase large subunit domains**		
pENTR221 CASP1 LS	PCR	this study
pENTR221 CASP2 LS	PCR	this study
pENTR221 CASP3 LS	PCR	this study
pENTR221 CASP5 LS V217	PCR	this study
pENTR221 CASP6 LS	PCR	this study
pENTR221 CASP7 LS	PCR	this study
pENTR221 CASP8 LS	PCR	this study
pENTR221 CASP9 LS	PCR	this study
pENTR221 CASP10 LS	PCR	this study
**Caspase 5 domains**		
pENTR221 CASP5 P30 LR	synthetic DNA	this study
pENTR221 CASP5 P30 LG	synthetic DNA	this study
pENTR221 CASP5 P30 LE	synthetic DNA	this study
pENTR221 CASP5 P30 TE	synthetic DNA	this study
**OspC entry clones**		
pENTR223 OspC1		(1)
pENTR223 OspC2		(1)
pENTR223 OspC3		(1)
pENTR223 OspC1_F183A, F206A, D226A	synthetic DNA	this study
pENTR223 OspC2_F188A, F211A, D231A	synthetic DNA	this study
pENTR22s OspC3_F188A, F211A, D231A	synthetic DNA	this study
pENTR221 OspC2_A	overlap PCR	this study
pENTR221 OspC2_B	overlap PCR	this study
pENTR221 OspC2_C	overlap PCR	this study

pENTR221 OspC2_8	overlap PCR	this study
pENTR221 OspC3_A	overlap PCR	this study
pENTR221 OspC3_B	overlap PCR	this study
pENTR221 OspC3_C	overlap PCR	this study
pENTR221 OspC3_8	overlap PCR	
**OspC2 mutants**		
pENTR223 OspC2_5 NS (N407K M410R E413N E423KS425G)	synthetic DNA	this study
pENTR223 OspC2_4A NS (N407K M410R E413N E423K)	synthetic DNA	this study
pENTR223 OspC2_4B NS (M410R E413N E423KS425G)	synthetic DNA	this study
pENT221 OspC2_3A NS (N407K M410R E413N)	synthetic DNA	this study
pENTR223 OspC2_3B NS (M410R E413N E423K)	synthetic DNA	this study
pENTR221 OspC2_2 NS (M410R E413N)	synthetic DNA	this study
pENTR223 OspC2_1 NS (M410R)	synthetic DNA	this study
**OspC3 mutants**		
pENTR221-OspC3_3A NS (K407N R410M N413E)	synthetic DNA	this study
pENTR221-OspC3_3B NS (R410M N413E K423E)	synthetic DNA	this study
pENTR221 OspC3_2 NS (R410M N413E)	synthetic DNA	this study
pENTR223 OspC3_1 NS (R410M)	synthetic DNA	this study

TABLE S2 Expression and miscellaneous plasmids

name	Description	source	ref
**DESTINATION PLASMIDS**			
pGBKT7-GW	GAL4BD-ccdB, yeast 2 micron	Addgene 61703	(2)
pGADT7-GW	GAL4AD-ccdB, yeast 2 micron	Addgene 61702	(2)
pDSW206-ccdB-3xFLAG	pTRC (IPTG), ccdB-3xFLAG, low copy (ColE1 ori), AMP^R		(1)
pC1-eGFP-ccdB	pCMV eGFP-ccdB		(3)
**YEAST EXPRESSION PLASMIDS**			
**Binding domain fusions**			
pGBKT7 OspC2	pGPD-GAL4BD-OspC2	GW	this work
pGBKT7 OspC3	pGBD-GAL4BD-OspC3	GW	this work
pGBKT7 OspC2_A	pGBD-GAL4BD-OspC2_A	GW	this work
pGBKT7 OspC2_B	pGBD-GAL4BD-OspC2_B	GW	this work
pGBKT7 OspC2_C	pGBD-GAL4BD-OspC2_C	GW	this work
pGBKT7 OspC3_A	pGBD-GAL4BD-OspC3_A	GW	this work
pGBKT7 OspC3_B	pGBD-GAL4BD-OspC3_B	GW	this work
pGBKT7 OspC3_C	pGBD-GAL4BD-OspC3_C	GW	this work
**Activation domain fusions**			
pGAD CASP1	pGPD-GAL4AD-CASP1	GW	this work
pGAD CASP2	pGPD-GAL4AD-CASP2	GW	this work
pGAD CASP3	pGPD-GAL4AD-CASP3	GW	this work
pGAD CASP4	pGPD-GAL4AD-CASP4	GW	this work
pGAD CASP5	pGPD-GAL4AD-CASP5	GW	this work
pGAD CASP6	pGPD-GAL4AD-CASP6	GW	this work
pGAD CASP7	pGPD-GAL4AD-CASP7	GW	this work
pGAD CASP8	pGPD-GAL4AD-CASP8	GW	this work
pGAD CASP9	pGPD-GAL4AD-CASP9	GW	this work
pGAD CASP10	pGPD-GAL4AD-CASP10	GW	this work
pGAD CASP4 CARD	pGPD-GAL4AD-CASP4_CARD	GW	this work
pGAD CASP4 S	pGPD-GAL4AD-CASP4_S	GW	this work
pGAD CASP1 L	pGPD-GAL4AD-CASP1_L	GW	this work
pGAD CASP2 L	pGPD-GAL4AD-CASP2_L	GW	this work
pGAD CASP3 L	pGPD-GAL4AD-CASP3_L	GW	this work
pGAD CASP4 L	pGPD-GAL4AD-CASP4_L	GW	this work
pGAD CASP5 L	pGPD-GAL4AD-CASP5_L	GW	this work
pGAD CASP6 L	pGPD-GAL4AD-CASP6_L	GW	this work
pGAD CASP7 L	pGPD-GAL4AD-CASP7_L	GW	this work

pGAD CASP8 L	pGPD-GAL4AD-CASP8_L	GW	this work
pGAD CASP9 L	pGPD-GAL4AD-CASP9_L	GW	this work
pGAD CASP10 L	pGPD-GAL4AD-CASP10_L	GW	this work
pGAD CASP4 L-S	pGPD-GAL4AD-CASP4_L-S	GW	this work
pGAD CASP5 L-S_LR	pGPD-GAL4AD-CASP5_L-S_LR	GW	this work
pGAD CASP5 L-S_VR	pGPD-GAL4AD-CASP5_L-S_VR	GW	this work
pGAD CASP5 L-S_LE	pGPD-GAL4AD-CASP5_L-S_LE	GW	this work
pGAD CASP5 L-S_LG	pGPD-GAL4AD-CASP5_L-S_LG	GW	this work
pGAD CASP5 L-S_TE	pGPD-GAL4AD-CASP5_L-S_TE	GW	this work
**BACTERIAL EXPRESSION PLASMIDS**			
pDSW206_EV	no insert		(4)
pDSW206 OspC1	pTRC-OspC1-3xFLAG		(1)
pDSW206 OspC2	pTRC-OspC2-3xFLAG		(1)
pDSW206 OspC3	pTRC-OspC3-3xFLAG		(1)
pDSW206 OspC1_FFD	pTRC-OspC1_FFD-3xFLAG	Gibson	this work
pDSW206 OspC2_FFD	pTRC-OspC2_FFD-3xFLAG	Gibson	this work
pDSW206 OspC3_FFD	pTRC-OspC3_FFD-3xFLAG	Gibson	this work
**Chimeras**			
pDSW206 OspC2_A	pTRC-OspC2_A-3xFLAG	GW	this work
pDSW206 OspC2_B	pTRC-OspC2_B-3xFLAG	GW	this work
pDSW206 OspC2_C	pTRC-OspC2_C-3xFLAG	GW	this work
pDSW206 OspC2_8	pTRC-OspC2_N407K M410R E413N E423K S425G S436N R445K A449V-3xFLAG	GW	this work
pDSW206 OspC3_A	pTRC-OspC3_A-3xFLAG	GW	this work
pDSW206 OspC3_B	pTRC-OspC3_B-3xFLAG	GW	this work
pDSW206 OspC3_C	pTRC-OspC3_C-3xFLAG	GW	this work
pDSW206 OspC3_8	pTRC-OspC3_K407N R410M N413E K423E G425S N436S K445R V449A-3xFLAG	GW	this work
**OspC2 point mutations**			
pDSW206 OspC2_5	pTRC-OspC2_N407K M410R E413N E423K S425G-3xFLAG	GW	this work
pDSW206 OspC2_4A	pTRC-OspC2_N407K M410R E413N E423K-3xFLAG	GW	this work
pDSW206 OspC2_4B	pTRC-OspC2_ M410R E413N E423K S425G -3xFLAG	GW	this work
pDSW206 OspC2_3A	pTRC-OspC2_N407K M410R E413N-3xFLAG	GW	this work
pDSW206 OspC2_3B	pTRC-OspC2_M410R E413N E423-3xFLAG	GW	this work
pDSW206 OspC2_2	pTRC-OspC2_M410R E413N-3xFLAG	GW	this work
pDSW206 OspC2_1	pTRC-OspC2_M410R-3xFLAG	GW	this work
**OspC3 point mutations**			
pDSW206 OspC3_3A	pTRC-OspC3_K407N R410M N413E-3xFLAG	GW	this work

pDSW206 OspC3_3B	pTRC-OspC3_R410M N413E K423E -3xFLAG	GW	this work
pDSW206 OspC3_2	pTRC-OspC3_ R410M N413E-3xFLAG	GW	this work
pDSW206 OspC3_1	pTRC-OspC3_R410M -3xFLAG	GW	this work
**MAMMALIAN EXPRESSION PLASMIDS**			
pC1-eGFP			Clontech
pC1-eGFP-OspC1	pCMV-eGFP		(5)
pC1-eGFP-OspC2	pCMV-eGFP-OspC1		(5)
pC1-eGFP-OspC3	pCMV-eGFP-OspC2		(5)
pC1-eGFP-OspC1_FFD	pCMV-eGFP-OspC3	Quick Change	this work
pC1-eGFP-OspC2_FFD	pCMV-eGFP-OspC1_F183A, F206A, D226A	Quick Change	this work
pC1-eGFP-OspC3_FFD	pCMV-eGFP-OspC2_F188A, F211A, D231A	Quick Change	this work
pC1-eGFP-OspC2_4A	pCMV-eGFP-OspC2_N407K M410R E413N E423K	GW	this work
pC1-eGFP-OspC3_4A	pCMV-eGFP-OspC3_K407N R410M N413E K423E	Gibson	this work
pCMV6-CASP4*-FLAG	pCMV-CASP4_C258A-3xFLAG		gift from Sunny Shin
pCMV6-CASP5*-FLAG	pCMV Casp 5a (V217 R221) C315A-3xFLAG		gift from Sunny Shin
**MISCELLANEOUS PLASMIDS**			
pKD46	temperature sensitive, λ Red recombinase, AMP^R		(6)
pTKred	temperature sensitive, λ Red recombinase, SceI, $Spec^R$		(7)
pTKIP-tetA	PCR template for amplification of the 1.3 kb ‘landing pad’, R6K ori, AMPR, TETR		(7)
pCP20	temperature sensitive, AMP^R, FLP recombinase		(6)
pRK2073	helper plasmid, SpecR		(8)
pT3SS3.1	pLLX13 that carries T3SS genes from pVP LP-ΔipaJ::FRT-KAN^R-FRT (ipaJ::FRT-KanR-FRT thru orf131b)-LP, incP ori, TET^R, KAN^R		(9)
pBAD33_AfaI	AfaI expression vector, 15A ori, CmR		(10)
pBR322_AfaI	AfaI expression vector, ColE1 ori, AmpR		(11)

Table S3 **Bacterial and yeast strains**

**Yeast**		
MaV103	*MAT**a***, leu2-3,112, trp1-901, his3-Δ200, ura3-52, gal4Δ, gal80Δ, cyh2[r], can1[r], GAL1::HIS3@LYS2, GAL1::LacZ, SPAL10::URA3@ura3.	(12)
MaV203	*MAT*α, leu2-3,112, trp1-901, his3-Δ200, ura3-52, gal4Δ, gal80Δ, cyh2[r], can1[r], GAL1::HIS3@LYS2, GAL1::LacZ, SPAL10::URA3@ura3.	(12)
Shigella flexneri		
Shigella flexneri		(13)
*S. flexneri/*AfaI	*S. flexneri* 2457T 2a, pBR322-AfaI	this study
BS103	*S. flexneri* 2457T 2a, virulence plasmid cured	(14)
BS103--LPatp/gid	BS103	this study
mT3Sf		this study
mT3SfΔ*mxiE*		this study
Δ*ospC2 Shigella*	*S. flexneri* 2457T Δ*ospC2*::KanR, pBR322-AfaI	this study
Δ*ospC3 Shigella*	*S. flexneri* 2457T Δ*ospC3*::KanR, pBR322-AfaI	(9)
**mT3Sf**		
mT3Sf	mT3SfΔ*mxiE*	this study
mT3Sf::EV	mT3SfΔ*mxiE*: pNG12 VirB, pBAD33-AfaI, pDSW206_EV	this study
mT3Sf::OspC1	mT3SfΔ*mxiE*: pNG12 VirB, pBAD33-AfaI, pDSW206_OspC1	this study
mT3Sf::OspC2	mT3SfΔ*mxiE*: pNG12 VirB, pBAD33-AfaI, pDSW206_OspC2	this study
mT3Sf::OspC3	mT3SfΔ*mxiE*: pNG12 VirB, pBAD33-AfaI, pDSW206_OspC3	this study
mT3Sf::OspC1_FFD	mT3SfΔ*mxiE*: pNG12 VirB, pBAD33-AfaI, pDSW206_OspC1_FFD	this study
mT3Sf::OspC2_FFD	mT3SfΔ*mxiE*: pNG12 VirB, pBAD33-AfaI, pDSW206_OspC2_FFD	this study
mT3Sf::OspC3_FFD	mT3SfΔ*mxiE*: pNG12 VirB, pBAD33-AfaI, pDSW206_OspC3_FFD	this study
mT3Sf::OspC2_1	mT3SfΔ*mxiE*: pNG12 VirB, pBAD33-AfaI, pDSW206_OspC2_1	this study
mT3Sf::OspC2_2	mT3SfΔ*mxiE*: pNG12 VirB, pBAD33-AfaI, pDSW206_OspC2_2	this study
mT3Sf::OspC2_3A	mT3SfΔ*mxiE*: pNG12 VirB, pBAD33-AfaI, pDSW206_OspC2_3A	this study
mT3Sf::OspC2_3B	mT3SfΔ*mxiE*: pNG12 VirB, pBAD33-AfaI, pDSW206_OspC2_3B	this study
mT3Sf::OspC2_4A	mT3SfΔ*mxiE*: pNG12 VirB, pBAD33-AfaI, pDSW206_OspC2_4A	this study
mT3Sf::OspC2_4B	mT3SfΔ*mxiE*: pNG12 VirB, pBAD33-AfaI, pDSW206_OspC2_4B	this study

mT3Sf::OspC2_5	mT3SfΔ*mxiE*: pNG12 VirB, pBAD33-AfaI, pDSW206_OspC2_5	this study
mT3Sf::OspC2_8	mT3SfΔ*mxiE*: pNG12 VirB, pBAD33-AfaI, pDSW206_OspC2_8	this study
mT3Sf::OspC3_1	mT3SfΔ*mxiE*: pNG12 VirB, pBAD33-AfaI, pDSW206_OspC3_1	this study
mT3Sf::OspC3_2	mT3SfΔ*mxiE*: pNG12 VirB, pBAD33-AfaI, pDSW206_OspC3_2	this study
mT3Sf::OspC3_3A	mT3SfΔ*mxiE*: pNG12 VirB, pBAD33-AfaI, pDSW206_OspC3_3A	this study
mT3Sf::OspC3_3B	mT3SfΔ*mxiE*: pNG12 VirB, pBAD33-AfaI, pDSW206_OspC3_3B	this study
mT3Sf::OspC3_8	mT3SfΔ*mxiE*: pNG12 VirB, pBAD33-AfaI, pDSW206_OspC3_8	this study
Δ*ospC3 Shigella*/EV	Δ*ospC3 Shigella,*pBR322-AfaI, pDSW206_EV	this study
Δ*ospC3 Shigella*/OspC2	Δ*ospC3 Shigella,*pBR322-AfaI, pDSW206_EV	this study
Δ*ospC3 Shigella*/OspC3	Δ*ospC3 Shigella,*pBR322-AfaI, pDSW206_OspC3	this study
Δ*ospC3 Shigella*/OspC2_1	Δ*ospC3 Shigella,*pBR322-AfaI, pDSW206_OspC2_1	this study
Δ*ospC3 Shigella*/OspC2_2	Δ*ospC3 Shigella,*pBR322-AfaI, pDSW206_OspC2_2	this study
Δ*ospC3 Shigella*/OspC2_3A	Δ*ospC3 Shigella,*pBR322-AfaI, pDSW206_OspC2_3A	this study
Δ*ospC3 Shigella*/OspC2_3B	Δ*ospC3 Shigella,*pBR322-AfaI, pDSW206_OspC2_3B	this study
Δ*ospC3 Shigella*/OspC2_4A	Δ*ospC3 Shigella,*pBR322-AfaI, pDSW206_OspC2_4A	this study
Δ*ospC3 Shigella*/OspC2_4B	Δ*ospC3 Shigella,*pBR322-AfaI, pDSW206_OspC2_4B	this study
Δ*ospC3 Shigella*/OspC2_5	Δ*ospC3 Shigella,*pBR322-AfaI, pDSW206_OspC2_5	this study
Δ*ospC3 Shigella*/OspC2_8	Δ*ospC3 Shigella,*pBR322-AfaI, pDSW206_OspC2_8	this study

REFERENCES

1. Schmitz AM, Morrison MF, Agunwamba AO, Nibert ML, Lesser CF. 2009. Protein interaction platforms: visualization of interacting proteins in yeast. Nat Methods 6:500–502.
2. Lu Q, Tang X, Tian G, Wang F, Liu K, Nguyen V, Kohalmi SE, Keller WA, Tsang EWT, Harada JJ, Rothstein SJ, Cui Y. 2010. Arabidopsis homolog of the yeast TREX-2 mRNA export complex: components and anchoring nucleoporin. Plant J 61:259–270.
3. Sandstrom A, Mitchell PS, Goers L, Mu EW, Lesser CF, Vance RE. 2019. Functional degradation: A mechanism of NLRP1 inflammasome activation by diverse pathogen enzymes. Science 364:eaau1330.
4. Weiss DS, Chen JC, Ghigo J-M, Boyd D, Beckwith J. 1999. Localization of FtsI (PBP3) to the Septal Ring Requires Its Membrane Anchor, the Z Ring, FtsA, FtsQ, and FtsL. J Bacteriol 181:508–520.
5. Alphonse N, Wanford JJ, Voak AA, Gay J, Venkhaya S, Burroughs O, Mathew S, Lee T, Evans SL, Zhao W, Frowde K, Alrehaili A, Dickenson RE, Munk M, Panina S, Mahmood IF, Llorian M, Stanifer ML, Boulant S, Berchtold MW, Bergeron JRC, Wack A, Lesser CF, Odendall C. 2022. A family of conserved bacterial virulence factors dampens interferon responses by blocking calcium signaling. Cell 185:2354-2369.e17.
6. Datsenko KA, Wanner BL. 2000. One-step inactivation of chromosomal genes in *Escherichia coli* K-12 using PCR products. Proc Natl Acad Sci U S A 97:6640–6645.
7. Kuhlman TE, Cox EC. 2010. Site-specific chromosomal integration of large synthetic constructs. Nucleic Acids Res 38:e92.
8. Figurski DH, Helinski DR. 1979. Replication of an origin-containing derivative of plasmid RK2 dependent on a plasmid function provided in trans. Proc Natl Acad Sci U S A 76:1648–1652.
9. Mou X, Souter S, Du J, Reeves AZ, Lesser CF. 2018. Synthetic bottom-up approach reveals the complex interplay of *Shigella* effectors in regulation of epithelial cell death. Proc Natl Acad Sci U S A 115:6452–6457.
10. Yi C, Allen JE, Russo B, Lee SY, Heindl JE, Baxt LA, Herrera BB, Kahoud E, MacBeath G, Goldberg MB. 2014. Systematic analysis of bacterial effector-postsynaptic density 95/disc large/zonula occludens-1 (PDZ) domain interactions demonstrates *Shigella* OspE protein promotes protein kinase C activation via PDLIM proteins. J Biol Chem 289:30101–30113.
11. Labigne-Roussel AF, Lark D, Schoolnik G, Falkow S. 1984. Cloning and expression of an afimbrial adhesin (AFA-I) responsible for P blood group-independent, mannose-resistant hemagglutination from a pyelonephritic *Escherichia coli* strain. Infect Immun 46:251–259.
12. Walhout AJ, Vidal M. 2001. High-throughput yeast two-hybrid assays for large-scale protein interaction mapping. Methods 24:297–306.
13. Labrec EH, Schneider H, Magnani TJ, Formal SB. 1964. Epithelial cells penetration as an essential step in the pathogenesis of bacillary dysentery. J Bacteriol 88:1503–1518.

14. Maurelli AT, Blackmon B, Curtiss R. 1984. Loss of pigmentation in *Shigella flexneri* 2a is correlated with loss of virulence and virulence-associated plasmid. Infect Immun 43:397–401.
